# Clinical Trial Registration and Reporting: Drug Therapy and Prevention of Cardiac-Related Infections

**DOI:** 10.3389/fphar.2019.00757

**Published:** 2019-07-05

**Authors:** Lin-Lu Ma, Yang Qiu, Mei-Na Song, Yun Chen, Jian-Xin Qu, Bing-Hui Li, Ming-Juan Zhao, Xin-Can Liu

**Affiliations:** ^1^Department of Cardiology, First Affiliated Hospital of Henan University of Traditional Chinese Medicine, Zhengzhou, China; ^2^Center for Evidence-Based and Translational Medicine, Henan University of Traditional Chinese Medicine, Zhengzhou, China; ^3^Cardiovascular Department, Kaifeng Central Hospital, Kaifeng, China; ^4^Department of Nursing, Beijing Tsinghua Changgung Hospital, Beijing, China; ^5^Center for Evidence-Based Medicine, Institute of Evidence-Based Medicine and Knowledge Translation, Henan University, Kaifeng, China; ^6^Department of Cardiology, The First Affiliated Hospital of Henan University, Kaifeng, China

**Keywords:** cardiac-related infections, Clinicaltrials.gov, clinical trials, trial registration, antibiotics

## Abstract

**Objective:** Clinical trials are the source of evidence. ClinicalTrials.gov is valuable for analyzing current conditions. Until now, the state of drug interventions for heart infections is unknown. The purpose of this study was to comprehensively assess the characteristics of trials on cardiac-related infections and the status of drug interventions.

**Methods:** The website ClinicalTrials.gov was used to obtain all registered clinical trials on drug interventions for cardiac-related infections as of February 16, 2019. All registration studies were collected, regardless of their recruitment status, research results, and research type. Registration information, results, and weblink-publications of those trials were analyzed.

**Results:** A total of 45 eligible trials were evaluated and 86.7% of them began from or after 2008 while 91.1% of them adopted interventional study design. Of all trials, 35.6% were completed and 15.6% terminated. Besides, 62.2% of interventional clinical trials recruited more than 100 subjects. Meanwhile, 86.7% of the eligible trials included adult subjects only. Of intervention trials, 65.8% were in the third or fourth phase; 78.1% adopted randomized parallel assignment, containing two groups; 53.6% were masking, and 61.0% described treatment. Moreover, 41.5% of the trials were conducted in North America while 29.3% in Europe. Sponsors for 40.0% of the studies were from the industry. Furthermore, 48.9% of the trials mentioned information on monitoring committees, 24.4% have been published online, and 13.3% have uploaded their results. Drugs for treatments mainly contained antibiotics, among which glycopeptides, β-lactams, and lipopeptides were the most commonly studied ones in experimental group, with the former ones more common. Additionally, 16.2% of the trials evaluated new antimicrobials.

**Conclusions:** Most clinical trials on cardiac-related infections registered at ClinicalTrials.gov were interventional randomized controlled trials (RCTs) for treatment. Most drugs focused in trials were old antibiotics, and few trials reported valid results. It is necessary to strengthen supervision over improvements in results, and to combine antibacterial activity with drug delivery regimens to achieve optimal clinical outcomes.

## Introduction

Infectious diseases are caused by microorganisms (bacteria, viruses, fungi, parasites, etc.) releasing toxins or invading body tissues due to patients’ poor constitution and insufficient resistance to pathogens (Friedrich, 2019). These diseases gravely threaten human health, and appropriate drug treatment represents an important management strategy (Zumla et al., 2016). With increasing pressure of anti-infective drug selection, the spread of bacterial resistance and the slowdown of developing new drugs, many previously treatable infectious diseases have now become “incurable” (Hughes, 2014). The problem of bacterial resistance is becoming a serious threat to global public health. An estimated 162,000 people die of multidrug-resistant infections in the United States each year (Burnham et al., 2019). The diagnosis and treatment of infectious diseases are constantly facing new challenges. What are the current status and challenges in the prevention and treatment of common infectious heart diseases and surgical infections in cardiac disease field?

Cardiac-related infections include cardiac infectious diseases, cardiac device infections [permanent pacemakers (PPMS) and implantable cardioverter defibrillators (ICD)], and heart surgery-related infections (Fong, 2009). Many studies have shown microbial infections exhibit many pathogenic behaviors in cardiac-related infections, especially in bacterial and viral infections, which can directly lead to infective endocarditis (IE), myocarditis, pericarditis, cardiac device (permanent pacemakers, and implantable cardioverter defibrillators) implantation infections, and cardio-surgery infections (Fowler et al., 2006; Bennett-Guerrero et al., 2010; Mentzelopoulos et al., 2013; Mayosi et al., 2014; Morillo et al., 2015; Iversen et al., 2019). Many experts have devoted to developing relevant diagnosis and treatment principles and plans for antibiotic prevention (Thornhill et al., 2018). Guidelines released by the European Heart Association and the American Heart Association recommend patients with infective endocarditis on the left side of the heart to accept intravenous antibiotic therapy for 6 weeks (Baddour et al., 2015; Habib et al., 2015). Intravenous therapy during long-term hospitalization may increase the risk of complications, while shorter hospitalization is associated with better outcomes (Boucher, 2019). Besides, the incidence of right-side IE is increasing due to repaired congenital heart disease, the applying of injectable drugs, as well as the implantation of more cardiac devices including cardiac pacemakers, implantable cardioverters, and resynchronization devices (Chirouze et al., 2015). Therefore, given changes in pathogen spectrum and threats from antibiotic resistance, exploring better clinical diagnosis and treatment strategies remains necessary (Nadji et al., 2005).

Clinical trials can provide valid evidence for the safety and efficacy of prevention, diagnosis, and treatment strategies (Califf et al., 2012). ClinicalTrials.gov is a clinical trial database jointly run by the National Library of Medicine (NLM) and the US Food and Drug Administration (FDA) under the National Institutes of Health (NIH). In European Union and the United States, registering all interventional clinical trials is mandatory (Zarin et al., 2011). The International Committee of Medical Journal Editors (ICMJE) announced a policy in which the registration of clinical trials is stipulated to be a precondition for publication (De Angelis et al., 2005). ClinicalTrials.gov is the largest clinical trial registry, with high weekly growth rates for new entries, detailed information on past and present clinical trials, and high transparency and accessibility. Therefore, it could offer even more trials-obtained details than those reported in final peer-reviewed publications (Cihoric et al., 2017).

In recent years, with changes in pathogen spectrum and growing threats from antibiotic resistance, rational use of drugs faces challenges. Therefore, we limited our current analysis to clinical trials accessible at ClinicalTrials.gov to assess the characteristics of cardiac-related infection trials and the status of drug interventions.

## Methods

Accessible records of all clinical trials registered at ClinicalTrials.gov were downloaded, using its advanced search function to search for the terms “cardiac disease, infection,” “endocarditis,” “pericarditis,” “myocarditis,” “coronary artery, infection,” “aortitis,” and “rheumatic heart disease” respectively for “condition or disease” on February 16, 2019. All types of studies were incorporated, including interventional (clinical trials), observational, and expanded studies. Trials of both open (not yet being recruited, recruited) and closed (enrolled through invitation; active, not recruited; suspended; terminated; completed; withdrawn; unknown status) statuses were considered for inclusion. No restrictions were imposed on study results or their enrolled patients’ age. All diseases interested in study must be exactly caused by pathogenic microorganism. All included clinical trials must have definitive records on identified anti-infective drugs.

All the following information was extracted from each study: tracking information: actual start date of the study; descriptive information: study type, study phase, study design: interventional study (allocation, intervention model, masking, and primary purpose) and observational study (model, time perspective), number of arms, trial medications, study result, and online linked publications; recruitment information: recruitment status, actual enrollment, estimated completion date of the study, sex/gender, ages, and location; administrative information: National Clinical Trial (NCT) number, data on monitoring committee (DMC), primary study sponsor, collaborators, and funder type.

All trials were then further subdivided according to classification entry. Descriptive statistics were used to describe qualitative results. Percentage frequency distributions were adopted for categorical data.

## Results

As of February 16, 2019, 297, 86, 21, 46, 42, 6, and 31 registered trials were identified on clinicaltrials.gov, using the terms “cardiac disease, infection,” “endocarditis,” “pericarditis,” “myocarditis,” “coronary artery, infection,” “aortitis,” and “rheumatic heart disease,” respectively. We excluded duplicated studies and those using non-anti-infective drugs during initial review. In addition, we confirmed that each of the included studies focused diseases directly caused by the infection of pathogenic microorganisms during manual review process. After excluding 484 trials, 45 trials were eventually included (23 focusing on infective endocarditis, three on Chagas heart disease, two on coronary infection, one on parvovirus-mediated cardiomyopathy, one on tuberculous pericarditis, one on children with rheumatic heart disease, 1 on post-resuscitation infection after cardiac arrest and 13 on heart-related device/surgical infections, see **Supplementary Table S1**).

## General Characteristics of the Included Clinical Trials

The enrolled trials were registered between 1999 and 2019, and most (86.7%) of them began between 2008 and 2019. Study duration was within 36 months in more than half of the trials (62.2%), between 36 and 72 months in 26.7% of the trials, and more than 72 months in 11.1% of the trials. Of the eligible trials, 41 (91.1%) were intervention trials and the other four (8.9%) were observational trials. Completed status was dominant in the included trials (*n* = 16, 35.6%), followed by recruiting status (*n* = 11, 24.5%). Seven trials (15.6%) were terminated (three lacking funds; two lacking statistical power; one due to business reasons; one due to expired commitment) and one was withdrawn (unable to recruit patients within specified time period. No patients had been enrolled in the study). Most trials actually enrolled a large number of participants; specifically, 62.2% recruited 100 or more participants, 20.0% more than 1,000 participants, and one even recruited 4,000 participants. The included trials were mainly focused on adult patients, that is, 39 (86.7%) only included adult patients, three included individuals less than or equal to 18 years old, and two included subjects younger than 18 years old (**Table 1**).

**Table 1:** General characteristics of the included trials.

	Number	Percent
*Study start date*		
Prior to 2008	6	13.3
2008–2010	11	24.5
2011–2013	6	13.3
2014–2016	10	22.2
2017–2019	12	26.7
*Length of study time*		
0 < L ≤ 36m*	28	62.2
36m < L ≤ 72m	12	26.7
L > 72m	5	11.1
*Study type*		
Interventional	41	91.1
Observational	4	8.9
*Recruitment status*		
Not yet recruiting	3	6.7
Recruiting	11	24.5
Enrolling by invitation	1	2.2
Active, not recruiting	3	6.7
Terminated	7	15.6
Completed	16	35.6
Withdrawn	1	2.2
Unknown	3	6.7
*Actual enrollment*		
<100	17	37.8
100–1,000	17	37.8
1,000–2,000	7	15.6
≥2,000	2	4.4
NP	2	4.4
*Ages*		
<18 years	2	4.4
Up to 18 years	3	6.7
18 years and older	39	86.7
All	1	2.2
*Sex/gender*		
All	45	100.0

*m, month; NP, not provided.

## Methodological Quality of the Included Clinical Trials

Information about clinical trial phase was available in 38 out of 41 interventional studies. Besides, 16 (39.0%) trials belonged to phase 3, 11 (26.8%) to phase 4, and 8 (19.5%) to phase 2. There were 32 (78.1%) trials contained two groups, while six (14.6%) only 1 group. Most trials (78.1%) were randomized. Most commonly adopted intervention model was parallel assignment (*n* = 32, 78.1%), followed by single group assignment (*n* = 5, 12.2%). Almost half of the trials (46.4%) were not masked, eight (19.5%) were single masked, and other eight (19.5%) were quadruple masked. Main objectives of the interventional trials lay in treating (61.0%) and preventing (34.2%). Of the four observational studies, two (50.0%) were cohort studies; meanwhile, three (75.0%) trials were prospective and one (25.0%) retrospective (**Table 2**).

**Table 2:** Design data of the trials.

Study type		Number	Percent
Interventional (clinical trial)	*Trial phase*		
	Phase 1	2	4.9
	Phase 1/phase 2	1	2.5
	Phase 2	8	19.5
	Phase 3	16	39.0
	Phase 4	11	26.8
	NP	3	7.3
	*Number of arms*		
	1	6	14.6
	2	32	78.1
	3	1	2.4
	4	2	4.9
	*Allocation*		
	Non-randomized	3	7.3
	Randomized	32	78.1
	NP	6	14.6
	*Intervention model*		
	Single group assignment	5	12.2
	Parallel assignment	32	78.1
	Factorial assignment	1	2.4
	Crossover assignment	3	7.3
	*Masking (blinding)*		
	Open label	19	46.4
	Single	8	19.5
	Double	3	7.3
	Triple	2	4.9
	Quadruple	8	19.5
	NP (provided)	1	2.4
	*Primary purpose*		
	Treatment	25	61.0
	Prevention	14	34.2
	Health services research	1	2.4
	NP	1	2.4
Observational	*Observational model*		
	Cohort	2	50.0
	Other	1	25.0
	NP	1	25.0
	*Time perspective*		
	Prospective	3	75.0
	Retrospective	1	25.0

NP, not provided.

## Detailed Characteristics of the Included Clinical Trials

Among the 45 trials, 41 (91.1%) were conducted only on one continent, of which 17 (41.5%) were in North America, 12 (29.3%) in Europe, and six (14.6%) in Asia. Four trials (8.9%) were conducted on two or more continents, and one of them even involved individuals from four continents. Companies were listed as primary sponsors in 18 (40.0%) trials, universities in 11 (24.5%), and hospitals in six (13.3%). A small number of trials (28.9%) had collaborations, and one of them had nine collaborations. Most of the trials (64.5%) were supported by other-type funds, followed by industrial funds (24.4%). Less than half of the trials (48.9%) provided DMCs. Only six (13.3%) trials listed results at ClinicalTrials.gov. While 11 (24.4%) trials offered links to webpage publications displaying relevant results, and 10 (22.2%) were linked to PubMed citation through indexed NCT number of the studies. Most of the trials attached publications (72.7%) possessed more than two publications. Among the published trials, eight (72.7%) enjoyed an impact factor (IF) value no less than 5, and 27.3% no less than 40. (**Table 3**).

**Table 3:** Detailed characteristics of the included trials.

	Number	Percent
*Locations*		
Single continent	41	91.1
Asia	6	14.6
Europe	12	29.3
North America	17	41.5
South America	2	4.9
Africa	3	7.3
Oceania	1	2.4
Multiple continents	4	8.9
2 continents	3	75.0
>2 continents	1	25.0
*Study sponsor*		
University	11	24.5
Hospital	6	13.3
Industry	18	40.0
Other	10	22.2
*Collaborators*		
NP	32	71.1
Has collaborators	13	28.9
*Funder type*		
Other	29	64.5
Other/industry	4	8.9
Industry	11	24.4
Industry/U.S. Fed	1	2.2
*Data monitoring committee*		
Has data monitoring committee	22	48.9
Not have data monitoring committee	15	33.3
NP	8	17.8
*Study results*		
Has results	6	13.3
No results	39	86.7
*Publications of the study*		
No publications	34	75.6
Has publications	11	24.4
<2 publications	3	27.3
≥2 publications	8	72.7
IF of publications		
0 < IF < 5	3	27.3
5 ≤ IF < 10	2	18.2
10 ≤ IF < 40	0	0.0
IF ≥ 40	6	54.5

NP, not provided.

## Description of Drugs in the Included Clinical Trials

Experimental groups in all included trails involved 11 categories and 27 kinds of antibiotics. Of the 41 intervention trials, 37 compared for efficacy across different drugs, and four for different uses of same drugs (oral/intravenous antibiotics). Of the interventional trials, 25 investigated drugs for treatment, 15 for prevention, while one did not provide intervention targets. Among the trials on treatment, experimental group were mostly used to concentrate on antibiotics, three on anti-inflammatory drugs and one on vitamin supplement. Highly interested antibiotics in the trails were glycopeptides (*n* = 5), β-lactams (*n* = 5), and lipopeptides (*n* = 5). Daptomycin and vancomycin were most frequently discussed in experimental group. In control group, antibiotics appeared in all trials except in placebo/conventional treatment. β-Lactam antibiotics appeared most frequently, which included nine kinds of drugs. Vancomycin, gentamycin, and daptomycin were the most common single-drugs in control group. Among the trials on prevention, most experimental groups adopted antibiotics, two surgical area disinfectants and one probiotic. Most frequently applied antibiotics was glycopeptides (*n* = 4), and vancomycin represented the most commonly employed single-antibiotic. In control group, antibiotics and surgical area disinfectant were both focused on. Most frequently accepted antibiotics was β-lactams (*n* = 4), with cefazolin topping the list. In four observational studies, selected drugs mainly were antibiotics and hormones, and most popular antibiotics were quinolones. In addition, most trials (*n* = 31, 83.8%) evaluated old antibiotics, and six trials (16.2%) assessed new antimicrobials approved by the FDA in recent years (**Table 4**).

**Table 4:** Descriptions of drugs in trials.

Study type	Primary purpose	Experimental group			Comparison group		
		Drug type	Drug name	Frequency	Drug type	Drug name	Frequency
Interventional (clinical trial)	Treatment	*Antibiotic drugs*			*Antibiotic drugs*		
		Glycopeptides	Vancomycin	2	β-Lactams	Ceftriaxone	2
			Dalbavancin*	1		Amoxicillin	1
			Oritavancin*	1		Amicillin	1
			Telavancin	1		Penicillin G	1
		β-Lactams	Ceftriaxone	1		Cloxacillin	1
			Benzathine penicillin G	1		Oxacillin	1
			Imipenem	1		Semi-synthetic penicillin	1
			Amoxicillin	1		Synthetic penicillin	1
			Ceftobiprole medocaril*	1		Cefazolin	1
		Tetracycline	Doxycycline	1	Aminoglycosides	Gentamycin	4
		Macrolides	Azithromycin	1		Netilmicin	1
		Antifungals	Fluconazole	1	Glycopeptides	Vancomycin	7
		Quinolones	Levofloxacin	1	Lipopeptides	Daptomycin	3
		Lipopeptides	Daptomycin	5	Other antibiotics	Rifampicin	1
		Aminoglycosides	Gentamicin	1			
		Other antibiotics	Fosfomycin	1			
			CF-301*	1			
			Benznidazole	1			
			Rifabutin	1			
			Rifampicin	1			
		*Others*	Selenium	1			
			Immunoglobulins	1			
			Colchicine	1			
			Prednisolone	1			
	Prevention	Antibiotic drugs			Antibiotic drugs		
		Glycopeptides	Vancomycin	4	β-Lactams	Cefazolin	2
		β-Lactams	Cefazolin	2		Cephalexin	1
		Tetracycline	D-PLEX^*^	2		Ceftaroline	1
		Antivirals	Valganciclovir	2	Antivirals	Mycophenolate	1
			Mycophenolate	1	Quinolones	Levofloxacin	1
		Peptides	Polymyxin-B	1	Lincomycin	Clindamycin	1
			Bacitracin	1	Lipopeptides	Daptomycin	1
		Aminoglycosides	Gentamicin	1	Other antibiotics	Linezolid	1
		Other antibiotics	Mupirocin	1	*Others*	Chlorhexidine	1
		*Others*	Povidone iodine	1			
			Hydrogen peroxide	1			
			Synbiotic 2000	1			
	NP	Lipopeptides	Daptomycin	1			
Observational	Treatment	Antibiotic drugs					
		Quinolones	Trovafloxacin	1			
			Levofloxacin	1			
		Lipopeptides	CUBICIN	1			
		Other antibiotics	Fosfomycin	1			
		Others	Hydrocortisone	1			

*U.S. FDA-regulated Drug Product; NP, not provided.

## Description of Drugs in Varied Types of Cardiac-Related Infections

Drugs adopted for the prevention and treatment of cardiac-related infections were mainly concentrated on antibiotics, antivirals, and glucocorticoids. Antivirals were often employed in treating coronary infections, especially valganciclovir. Glycopeptides, β-lactams, and tetracycline antibiotics were often applied to prevent or treat infections related to open heart surgery; peptide antibiotics were for infections associated with implantations surgery. Lipopeptides were most commonly studied, followed by β-lactams and glycopeptides among the IE caused by *Staphylococci*. Glycopeptides, especially vancomycin, most commonly appeared in methicillin-resistant *Staphylococcus aureus* (MRSA)-induced IE studies, while β-lactams in those on IE caused by *Enterococcus faecalis*. Glucocorticoids were most adopted in heart diseases that cause systemic infections. In addition, there were also other non-antibiotics involved. In these studies, especially those related to IE, three drugs were adopted to explore effects of different modes of administration (oral *vs* intravenous treatment), and one drug for pharmacokinetic characteristics of different doses and frequency (**Table 5**).

**Table 5:** Description of drugs in varied types of cardiac-related infections.

Conditions/diseases		Experimental group			Comparison group		
		Antibiotic drugs	Drug name	F	Antibiotic drugs	Drug name	F
Coronary infection	Coronary heart disease/Chlamydophila pneumoniae infections	Tetracycline	Doxycycline	1			
		Macrolides	Azithromycin	1			
		Others	Rifabutin	1			
	Cardiac allograft vasculopathy/Cytomegalovirus infection	Antivirals	Valganciclovir	3	Antivirals	Pre-emptive mycophenolate	1
			Mycophenolate	1			
Cardiac surgery infection	Cardiac surgery/SSIs	Glycopeptides	Vancomycin	3	β-Lactams	Cefazolin	2
		β-Lactams	Cefazolin	2			
		Tetracycline	D-PLEX*	2			
		Aminoglycosides	Gentamicin	1			
		Antivirals	Valganciclovir	1			
		Others	Mupirocin	1			
		Non-antibiotic	Synbiotic 2000	1			
	Implantations surgery/infections	Non-antibiotic	Povidone iodine	1	Non-antibiotic	Chlorhexidine	1
			Hydrogen peroxide	1	β-Lactams	Cephalexin	1
		Others	Fluconazole	1	Linkes	Clindamycin	1
		Peptide antibiotics	Polymyxin B/bacitracin	1	Peptide antibiotics	Polymyxin B/bacitracin	1
					Quinolones	Levofloxacin	1
Infective endocarditis (IE)	IE/bacterial	Others	Fosfomycin	1			
	IE/microbial infection	Glycopeptides	Vancomycin	1	Aminoglycosides	Gentamycin	1
	IE/*Streptococci*, *Staphylococci* or *Enterococci* infecting		Antibiotic therapy^#^				
	IE/*Streptococcus-Enterococcus*	β-Lactams	Amoxicillin^#^	1	β-Lactams	Amoxicillin	1
						Ampicillin	1
						Penicillin G	1
						Ceftriaxone	1
					Aminoglycosides	Vancomycin	1
						Gentamicin	1
						Netilmicin	1
	IE/MRSA, *Streptococci*	Glycopeptides	Dalbavancin*	1			
	IE/MRSA	Glycopeptides	Vancomycin	1	Glycopeptides	Vancomycin	2
		Others	Fosfomycin	1			
		β-Lactams	Imipenem	1			
		Lipopeptides	Daptomycin	1			
	IE/SA	Lipopeptides	CUBICIN	1	Lipopeptides	Daptomycin	4
			Daptomycin	4			
		Quinolones	Levofloxacin^#^	2	Glycopeptides	Vancomycin	4
			Trovafloxacin	1	β-Lactams	Cloxacillin,	1
		Glycopeptides	Telavancin	1		Oxacillin,	1
			Vancomycin	1		Semi-synthetic Penicillin	1
						Synthetic penicillin	1
		β-Lactams	Ceftobiprole medocaril*	1		Cefazolin	1
		Aminoglycosides	Gentamici	1		Ceftaroline	1
		Others	CF-301*	1	Aminoglycosides	Gentamicine	2
			Rifampicin,1^#^	1	Others	Rifampicin	1
						Linezolid	1
	IE/*Enterococcus faecalis*	β-Lactams	Ceftriaxone^@^	1	β-Lactams	Ceftriaxone	1
		Lipopeptides	Daptomycin	1			
	IE/OUD	Glycopeptides	Oritavancin injection*	1			
			OPAT^#^	1			
Infectious myocarditis	Chagasic myocardiopathy/*Trypanosoma cruzi*	Others	Benznidazole	1			
		Non-antibiotic	Selenium	1			
			Colchicine	1			
	Myocardial diseases/ Parvovirus B19	Non-antibiotic	Intravenous Immunoglobulins	1			
Infectious valvulitis	RHD/group A *Streptococcus*	β-Lactams	Benzathine penicillin G	1			
Infectious pericarditis	Tuberculous pericarditis/HIV	Non-antibiotic	Prednisolone	1			
CA/infection	Post-resuscitation infection/infections	Non-antibiotic	Hydrocortisone	1			

*U.S. FDA-regulated Drug Product; ^#^the oral treatment vs IV treatment; ^@^Different doses of the same drug; F, frequency; SSIs, surgical site infections; MRSA, methicillin-resistant Staphylococcus aureus; SA, Staphylococcus aureus bacteria; OPAT, outpatient parenteral antibiotic therapy; OUD, opioid use disorder; RHD, rheumatic heart disease; HIV, human immunodeficiency virus; CA, cardiac arrest.

## Discussion

This study comprehensively analyzed drug trials registered on ClinicalTrials.gov, all of which explored the intervention of infectious heart diseases or cardiac-related surgery infections. Through the analysis, we found that the number of registered cardiac-related infectious diseases was less than that of infectious diseases related to other organs. In these trials, most took interventional design. One-third of the trials were completed and 15.6% terminated. Most intervention trials were in phase 3 or 4, randomized, parallel assignment, masking, and sufficient, and possessed large sample size. Meanwhile, 24.4% of the trials offered publication links accessing to study results, 13.3% uploaded their results, and less than half provided DMCs. Drugs for treatment were mainly antibiotics, with glycopeptides, β-lactams, and lipopeptides topping the list in experimental group, while glycopeptides dominated in experimental group among trails on prevention.

From the perspective of study design, the vast majority of the trials (78.1%) were randomized and parallel assignment with two arms. Randomization, an exceptionally powerful tool, largely prevents confusion and mitigates selection bias in treatment comparisons (Sessler and Imrey, 2015). Besides, 51.2% of the trials were blinded. Well-implemented masking simultaneously prevents measurement bias and placebo effects through balancing treatment effects (Devereaux and Yusuf, 2003). Meanwhile, 62.2% of the trials contained more than 100 participants, and 20.0% more than 1,000. Sample size affects many factors, like statistical power, effect size, population mean, and variance (Allareddy et al., 2014). Due to the lack of information on the ClinicalTrials.gov Registry, we failed to make accurate judgments. However, larger sample size can increase the accuracy of estimated treatment outcome in trail and results’ credibility (Ruberg and Akacha, 2017). The standardization of clinical study design is important in successfully implementing clinical research. Appropriate randomization method, adequate masking, and treatment assignment, selecting active comparator and reasonable target sample size are essential in realizing reliable (unbiased) treatment comparison (Pocock et al., 2015). But many trials did not mention specific procedures for randomization, allocation concealment, or the phase of open label. Therefore, we could not determine whether they possessed high quality. Additionally, 37.8% of the trials spanned more than 36 months, and 11.1% even lasted beyond 72 months. As for study time prolonging, time-dependent bacterial resistance rate would become a significant confounding factor (Wan et al., 2018). Because of varied study duration periods, bacterial resistance rates were different. When comparing drug efficacy, this aspect would possibly bias research conclusion (Venekamp et al., 2016). However, most trials chose random enrollment to alleviate the impact of this aspect. Of the trials, 17.8% were terminated or withdrawn, mainly due to inadequate enrollment, followed by lacking statistical power and business reasons. In addition, only 13.3% of the trials included children. The shortage of funds for children’s medication and insufficient drug development still represent major challenges facing clinical studies on children’s medications (Allegaert et al., 2018). We hope that the government can introduce relevant policies to encourage more research institutions to conduct drug trials on children to establish optimal clinical treatment strategies.

The selected trails were implemented in six continents, and 8.9% of them were conducted in two or more continents. As shown on the official website, ClinicalTrials.gov is a database of global clinical studies receiving both private and public funds. It covers a wide geographical range, making us accessible to more comprehensive information on diseases and more reliable results. Of the eligible trails, 28.9% had collaborators. Large-scale multicenter trials, exceeding single-center ones, would facilitate the recruitment of enough patients, speed trials’ progress (Brophy, 2015), and improve research’s external validity (Allareddy et al., 2014). However, to reduce the bias in study implementation, it is necessary for researchers in different institutions to accept uniform standards and to reach consistent understanding (American Society of Clinical Oncology, 2003). Besides, 48.9% of the trials offered DMCs; while DMC is vital in maintaining scientific integrity in trials, the authenticity and accuracy of trial data, and the safety of studied participants (Filippatos et al., 2017). Only 13.3% of the trials showed results on ClinicalTrials.gov, partly due to the presence of various extensions and exemptions. The Food and Drug Administration Amendments Act of 2007 (FDAAA) demands to submit “basic results” for certain types of clinical trials within 1 year after experiment completion (Phillips et al., 2017). Reporting study results is critical in advancing study progression and ensuring the safety of participants in clinical trials. Therefore, it is necessary to strengthen the supervision of online result publication (Lee et al., 2018). Among our selected trails, 24.4% offered web links to their publications with relevant results on ClinicalTrials.gov., of which 27.3% were published on top journals (IF ≥ 40). Good research design plays a crucial role in result publication (del Rio et al., 2014). At the same time, we found that most of the trials (66.7%) on top journals were funded by universities, hospitals, and research institutions, and industry-funded ones accounted for only one-third. Recent evidence indicates that trials funded by industry sources are likely to be biased in favor of sponsors’ products, thus causing obvious publication bias (Lundh et al., 2017).

In general, the vast majority of the trials focused on treating or preventing the occurrence of infection adopting antibiotics. Six trials (16.2%) evaluated new antimicrobials approved by the FDA in recent years. Since many pathogens are more resistant to existing antibiotics, it has become particularly important to develop new antibiotics to improve time-related bacterial resistance rates (Penchovsky and Traykovska, 2015). Two trials evaluated the efficacy and safety of different methods of using the same antibiotic. Given the global crisis of antimicrobial resistance, it is important to determine the optimal duration of applying intravenous and oral antibiotics and to form evidence-based recommendations about when to switch from intravenous to oral routes (McMullan et al., 2016).

Diseases in the selected trials involved infective endocarditis (IE), Chagas heart disease, coronary infection, parvovirus-mediated cardiomyopathy, tuberculous pericarditis, childhood rheumatic heart disease (RHD), and heart-related device/surgery infections. Options are limited for treating endocarditis caused by MRSA. Vancomycin is the standard treatment for blood infections caused by MRSA, but its effect is limited in treating endocarditis caused by MRSA. Its bactericidal activity is weaker than that of β-lactams, showing low permeability in the valves, while debates still exist on its applicability (del Rio et al., 2014). Many clinical trials have evaluated the efficiency of different types or doses of antibiotic treatments, such as daptomycin, CF-301, β-lactams, fosfomycin, dalbavancin, levofloxacin, and new glycopeptides (dalbavancin or oritavancin). Earlier researches also involved optimized antibiotics treatment options for IE patients with opioid use disorder (OUD), an infection that has recently doubled hospitalization rates. In addition, the effectiveness and safety of conversion from intravenous antibiotics to oral ones have also been well evaluated. The effect of Trypanosoma treatment on Chagas myocardiopathy caused by *T. cruzi* infection is still unclear. Benznidazole, colchicine, and selenium supplementation were included in the study to explore their clinical efficacy. Besides, two of our enrolled trials examined the effects of combined antibiotics or antiviral drugs on preventing changes in coronary vascular infections. In addition, an included trial investigated the effect of high-dose intravenous immunoglobulin on virus presence in patients with high load of parvovirus B19 in the heart. Tuberculous pericarditis is often accompanied by human immunodeficiency virus (HIV) infection, seeing poor prognosis, and a collected trial evaluated the efficacy and safety of adjunctive prednisolone therapy. Appropriate management for latent RHD has not been developed and no formal recommendations have been established. Some trials explored the prophylaxis and prognosis of intramuscular benzathine penicillin G (BPG). Despite the use of prophylactic systemic antibiotics, postoperative wound infections and infections associated with cardiac implantable electronic devices still represent serious threats after heart-related surgery (Mertz et al., 2011; Arnold and Chu, 2018), though many types of antibiotic treatment regimens have been compared and comprehensively analyzed. For example, when it comes to D-PLEX, vancomycin, mupirocin, etc., whether their applications should be directly intravenous, oral, topical, or through dressing during surgery has been constantly discussed.

According to published articles, patients with IE who received daptomycin treatment could reach similar outcomes to those treated with vancomycin, and such treatment could be used as a reasonable alternative (Fowler et al., 2006). The combination of fosfomycin with imipenem prevents drug resistance caused by single drug, and small doses can achieve ideal efficacy with reduced side effects among IE patients (del Rio et al., 2014). Fluoroquinolone combined with standard treatment does not improve treatment outcomes of *S. aureus bacteremia*, nor does it reduce mortality or the incidence of deep infection (Ruotsalainen et al., 2006). In patients with stable clinical conditions and sufficient response to initial treatment, transformation from intravenous administration to oral antibiotic treatment is not inferior to continued intravenous antibiotic treatment. Oral antibiotic may also minimize problems associated with outpatient parenteral treatment, logistics, and monitoring, and the risks of complications associated with intravenous catheters (e.g., local and systemic infections, and venous thrombosis) (Iversen et al., 2019). In a large randomized controlled trial, benznidazole significantly reduced the detection rate of parasitic infections in Chagas heart disease patients but did not significantly reduce the incidence of major clinical outcomes (Morillo et al., 2015). Adjunctive prednisolone had no significant effect on major clinical outcomes (Mayosi et al., 2014). Gentamicin-impregnated dressing had no significant effect on the rate of wound infection in patients undergoing open cardiac surgery (Bennett-Guerrero et al., 2010). Consequently, specific improvements in antibiotics, including the use of narrow-spectrum therapy, shortening treatment times, early transition from IV to oral therapy (Barlam et al., 2016), and the development of new drugs to prevent and treat infections, are key strategies in combating antimicrobial resistance.

The process of preventing and treating infectious diseases is complicated. Choosing right medication regimen, especially antibiotic regimen, requires a combination of the characteristics of pathogenic microorganism, the duration of medication application, adverse events caused by medication, and other outcomes (Cahill and Prendergast, 2016). For example, IE, one of the most common diseases associated with heart-related infectious diseases, is hard to be cured due to the characteristics of the infection itself, the bacterial species, and frequent comorbidities of the patients (del Rio et al., 2014; Schirone et al., 2018). A long treatment cycle (4–6 weeks of intravenously administered antibiotic agents) used to be required, and aminoglycoside often brings about side effect of nephrotoxicity (Baddour et al., 2015). In addition, the rate of antibiotic-resistant strains is increasing, which makes the establishment of effective antibiotic regimes more complicated. For this reason, some alternatives have been explored: Native valve endocarditis (NVE) was often treated with penicillin G and gentamicin for synergistic coverage of *Streptococci*. Patients with a history of intravenous drug use were treated with nafcillin and gentamicin to cover for methicillin-sensitive *Staphylococci*. The emergence of MRSA and penicillin-resistant *Streptococci* led to changes in empiric treatment, with liberal substitution of vancomycin in lieu of a penicillin antibiotic (Razmi and Magnusson, 2019). Treatment options range from in-hospital intravenous antibiotic therapy to partial oral therapy, replacement has minimized challenges associated with outpatient parental treatment (Boucher, 2019). Treatment offers the possibility of benefiting more patients, with multidisciplinary collaborative approach for the management of IE (Tan et al., 2018).

A recent review published in *Nature Reviews Immunology* (Parihar et al., 2019) presented a unique perspective on new drugs for the intervention of infectious diseases. As a host-directed treatment, statins wield powerful effects against infectious diseases caused by viruses, parasites, fungi, and bacteria. In particular, statin-mediated destructive effects, in combination with standard therapies, could interfere with microdomain lipids of MRSA, thus providing a novel anti-multidrug resistance infection strategy (Thangamani et al., 2015). However, we still need proof-of-concept clinical studies and large randomized controlled trials to verify the feasibility of statins acting as potential replacement therapy in infectious diseases.

Our research still had some limitations: 1) Clinical trials were obtained only from ClinicalTrials.gov, though this source contains most of global trials; and we might miss some trials registered in other 11 registries (Zarin et al., 2011) that were not fully evaluated. 2) Regarding to trial search and data extraction performed in this study, although all words supporting extensive research were used to maximize the number of included trials, some studies related to cardiac infection may not be found. 3) Since all information was obtained from ClinicalTrials.gov, intelligence not in this source was not further evaluated. At the same time, we only interpreted results acquired through ClinicalTrials.gov online links, and no more relevant publications were manually retrieved in the National Center for Biotechnology Information (NCBI) database using registration numbers. 4) Although final conclusions were relatively reliable based on publications with online links, considering that most of them were published in high-scoring journals, final establishment of clinical treatment methods need to take clinical therapies into account, after referring to more high-quality articles.

## Conclusions

Our study comprehensively analyzes the characteristics of trials registered at ClinicalTrials.gov for drug prevention or treatment of cardiac-related infections. Most clinical trials were interventional RCTs for treatment. While the majority of interested drugs were old antibiotics, and few trials reported valid study results. It is necessary to strengthen supervision over the improvement of trial results, and to implement explorations combining antibacterial activity with drug delivery regimens to achieve optimal clinical outcomes.

## Author Contributions

X-CL designed this study. L-LM and YQ performed the search and collected data, M-NS re-checked data. YC and J-XQ assessed and analyzed the data, and B-HL and M-JZ re-checked, assessed, and analyzed, L-LM and M-NS wrote the manuscript, X-CL reviewed the manuscript.

## Funding

This work was supported by the Program of Henan Province Traditional Chinese Medicine Research Project (grant No. 2017ZY1013) and the Special Research Project on the Construction of the National Traditional Chinese Medicine Clinical Research Base of the State Administration of Traditional Chinese Medicine of China (grant no. 2018JDZX008).

## Conflict of Interest Statement

The authors declare that the research was conducted without any commercial or financial relationships that could be construed as a potential conflict of interest.
